# Memphis Med. College

**Published:** 1850-01

**Authors:** 


*Memphis Medical College.*—We learn from a late No. of the Western Journal of Medicine and Surgery, that the organization of the Medical Institution recently established at Memphis, Tenn., has been broken up, and operations for the prerent, suspended.

